# Bis[(5-bromo­pyridin-2-yl)methano­lato-κ^2^ *N*,*O*]copper(II) monohydrate

**DOI:** 10.1107/S1600536813026974

**Published:** 2013-10-05

**Authors:** Tomohiko Hamaguchi, Issei Kawahara, Isao Ando

**Affiliations:** a Department of Chemistry, Faculty of Science, Fukuoka University, 8-19-1 Nanakuma, Jonan-ku, Fukuoka 814-0180, Japan

## Abstract

In the title compound, [Cu(C_6_H_5_BrNO)_2_]·H_2_O, the Cu^II^ ion has a square-planer N_2_O_2_ coordination environment. Slipped π–π stackings [centroid-centroid distances: 3.625 (3), 3.767 (3), 3.935 (3) and 4.255 (3) Å] between pyridine rings and Cu⋯π inter­actions (centroid-to-Cu^II^ distance: 3.56 Å) between Cu^2+^ ions and pyridine rings lead to a layered arrangement parallel to (010). Inter­molecular Br⋯O inter­actions [Br⋯O distances: 2.904 (3) and 3.042 (3) Å] and O—H⋯O hydrogen bonds form a three-dimensional network structure.

## Related literature

For bis(pyridin-2-ylmethanolato) complexes with four-coordinate Cu^II^, see: Antonioli *et al.* (2007 ▶); Boyle *et al.* (2010 ▶)
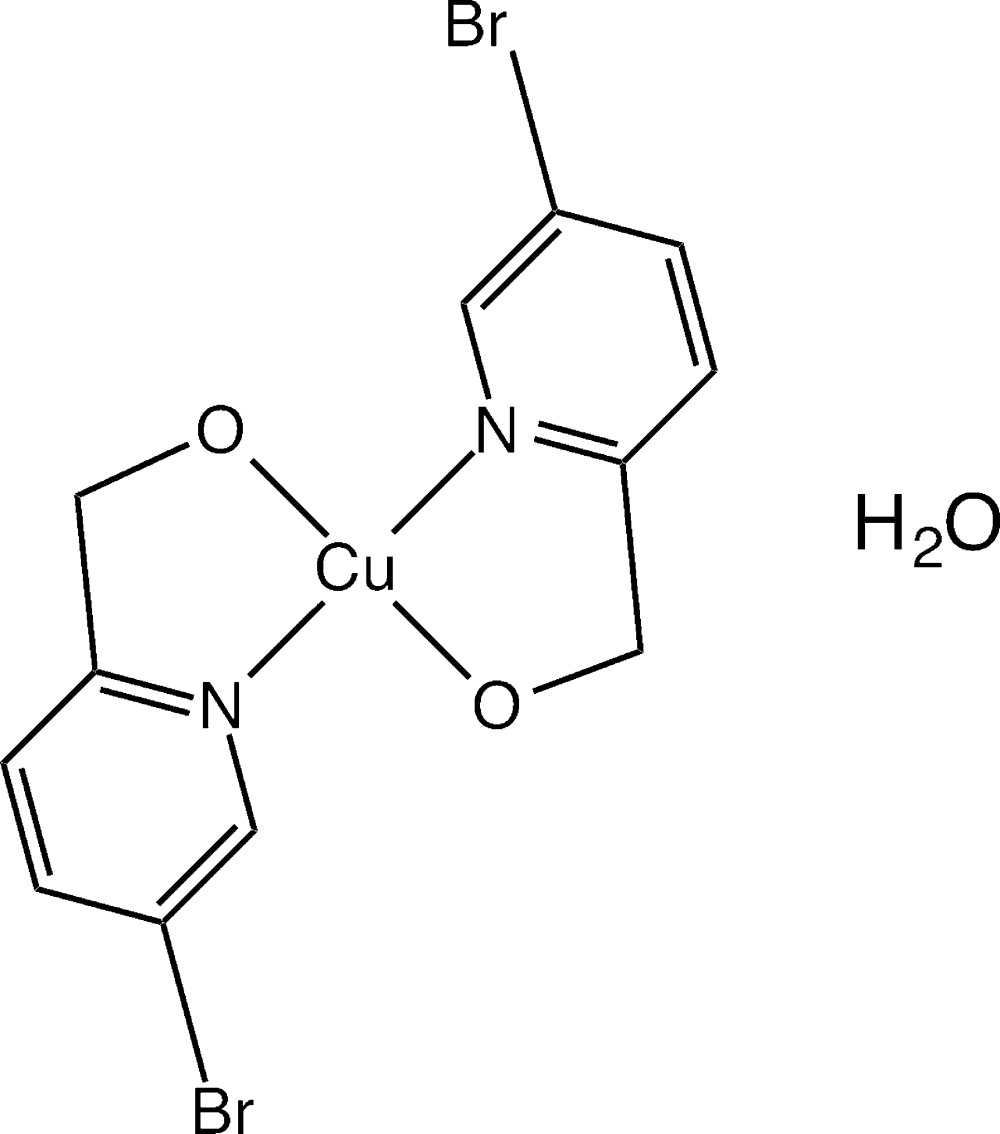


## Experimental

### 

#### Crystal data

[Cu(C_6_H_5_BrNO)_2_]·H_2_O
*M* *_r_* = 455.60
Triclinic, 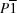

*a* = 7.1892 (9) Å
*b* = 7.5438 (9) Å
*c* = 13.2195 (15) Å
α = 99.338 (3)°
β = 103.334 (3)°
γ = 100.400 (3)°
*V* = 670.41 (14) Å^3^
*Z* = 2
Mo *K*α radiation
μ = 7.60 mm^−1^
*T* = 100 K
0.15 × 0.06 × 0.04 mm

#### Data collection

Rigaku R-AXIS RAPID diffractometer
Absorption correction: multi-scan (*ABSCOR*; Rigaku, 1995 ▶) *T*_min_ = 0.395, *T*_max_ = 0.751
6697 measured reflections
3074 independent reflections
2305 reflections with *I* > 2σ(*I*)
*R*_int_ = 0.039

#### Refinement

*R*[*F* ^2^ > 2σ(*F* ^2^)] = 0.028
*wR*(*F* ^2^) = 0.077
*S* = 1.20
3074 reflections
187 parameters
2 restraints
H atoms treated by a mixture of independent and constrained refinement
Δρ_max_ = 1.24 e Å^−3^
Δρ_min_ = −1.05 e Å^−3^

### 

Data collection: *RAPID-AUTO* (Rigaku, 2002 ▶); cell refinement: *RAPID-AUTO*; data reduction: *RAPID-AUTO*; program(s) used to solve structure: *SHELXS97* (Sheldrick, 2008 ▶); program(s) used to refine structure: *SHELXL97* (Sheldrick, 2008 ▶); molecular graphics: *Yadokari-XG 2009* (Wakita, 2001 ▶; Kabuto *et al.*, 2009 ▶), *Mercury* (Macrae *et al.*, 2006 ▶) and *ORTEP-3 for Windows* (Farrugia, 2012 ▶); software used to prepare material for publication: *Yadokari-XG 2009* and *publCIF* (Westrip, 2010 ▶).

## Supplementary Material

Crystal structure: contains datablock(s) I. DOI: 10.1107/S1600536813026974/ru2055sup1.cif

Structure factors: contains datablock(s) I. DOI: 10.1107/S1600536813026974/ru2055Isup2.hkl

Additional supplementary materials:  crystallographic information; 3D view; checkCIF report

## Figures and Tables

**Table 1:** Selected bond lengths (Å)

Cu1—O1	1.882 (3)
Cu1—O2	1.892 (3)
Cu1—N1	1.970 (3)
Cu1—N2	1.991 (3)

**Table 2:** Hydrogen-bond geometry (Å, °)

*D*—H⋯*A*	*D*—H	H⋯*A*	*D*⋯*A*	*D*—H⋯*A*
O3—H11⋯O1	0.80 (2)	1.95 (2)	2.740 (4)	170 (6)
O3—H12⋯O2^i^	0.82 (2)	2.01 (2)	2.825 (4)	171 (5)

Symmetry code: (i) 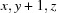.

**Fig. 1.**
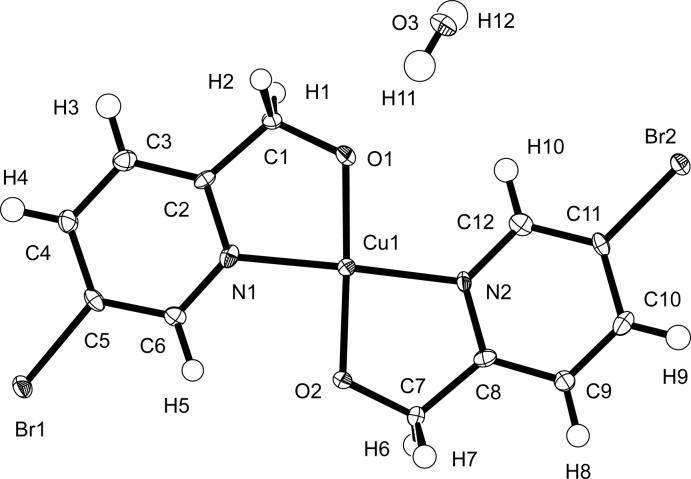
ORTEP drawing for the title complex with labeling showing 50% probability displacement ellipsoids.

**Fig. 2.**
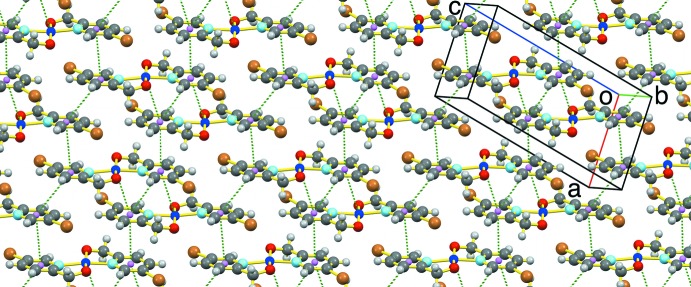
Crystal packing of the complex. Pale purple spheres indicate centroids of pyridine rings and green dashed lines indicate π-π or Cu···π interactions. H2O molecules are omitted for clarity. (blue: copper; red: oxygen; light blue: nitrogen; gray: carbon; brown: bromine; white: hydrogen)

**Fig. 3.**
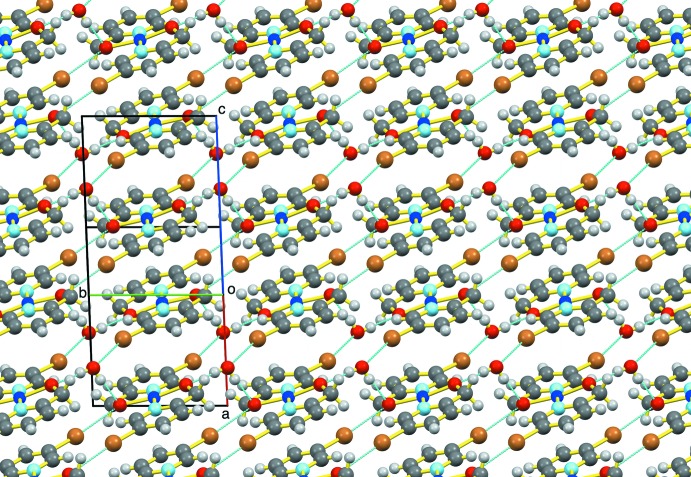
Crystal packing of the complex. Light blue dashed lines indicate Br···O halogen interaction or OH···O hydrogen bonding.

## supplementary crystallographic information

### 1. Comment

pyridin-2-ylmethanol is popular bidentate ligand, and many bis(pyridin-2-ylmethanolato) copper complexes are reported. The central copper ions of these complexes are mainly six- or five-coordinated; however, four-coordinated structure is few even in derivatives of pyridin-2-ylmethanol (Antonioli *et al.* (2007); Boyle *et al.* (2010)). Here, we report the crystal structure of [Cu^II^(5-bromo-pyridin-2-ylmethanolato)_2_]H_2_O which has a square planner Cu^II^ ion. As depicted in Fig. 1, the Cu^II^ ion is coordinated by two bidentated 5-bromo-pyridin-2-ylmethanolato ligands. A torsion angle of N1—O1—N2—O2 is -12.8 (1) ° and the Cu^II^ ion is located at -0.013 (2) Å from N1—O1—N2—O2 mean plane; therefore, the Cu^II^ has a slightly distorted square planer coordination environment. The complexes are connected *via* slipped π-π stackings between pyridine ring and pyridine ring [centroid-to-centroid distances: 3.625 (3), 3.767 (3), 3.935 (3) and 4.255 (3) Å; interplanar distances: 3.425 (2), 3.319 (2), 3.303 (2) and 3.594 (2) Å] and Cu···π interaction (centroid-to-Cu^II^ distance: 3.56 Å). These connections make this complex two-dimensional layered structure along with a c plane. (Fig. 2) Intermolecular Br···O halogen interaction [Br···O distances: 2.904 (3) and 3.042 (3) Å] and OH···O hydrogen bondings [O···O distances: 2.740 (4) and 2.825 (4) Å] make this two-dimensional layer to three-dimensional structure. (Fig. 3)

### 2. Experimental

A solution of triethylamine (0.125 mmol) in MeOH (0.5 ml) was added to a solution of 5-bromo-pyridin-2-ylmethanol (0.125 mmol) in MeOH (0.5 ml). A solution of CuSO_4_·5H_2_O (0.0625 mmol) in H_2_O (0.25 ml) was added to the mixture. After several hours, purple crystals crystallized from the purple solution.

### 3. Refinement

H atoms of OH were placed in a difference map and were refined coordinates only with restraints of O—H bond length (0.82 (2) Å) and *U*_iso_(H) = 1.5*U*_eq_(O). Other H atoms were placed at calculated positions and were treated as riding on the parent C atoms, with *U*_iso_(H) = 1.2*U*_eq_(C).

### Crystal data

**Table:**

[Cu(C_6_H_5_BrNO)_2_]·H_2_O	*V* = 670.41 (14) Å^3^
*M**_r_* = 455.60	*Z* = 2
Triclinic, *P*1	*F*(000) = 442
Hall symbol: -P 1	*D*_x_ = 2.257 Mg m^−^^3^
*a* = 7.1892 (9) Å	Mo *K*α radiation, λ = 0.71075 Å
*b* = 7.5438 (9) Å	µ = 7.60 mm^−^^1^
*c* = 13.2195 (15) Å	*T* = 100 K
α = 99.338 (3)°	Needle, purple
β = 103.334 (3)°	0.15 × 0.06 × 0.04 mm
γ = 100.400 (3)°	

### Data collection

**Table:**

Rigaku R-AXIS RAPID diffractometer	2305 reflections with *I* > 2σ(*I*)
Graphite monochromator	*R*_int_ = 0.039
ω scans	θ_max_ = 27.5°, θ_min_ = 3.2°
Absorption correction: multi-scan (*ABSCOR*; Rigaku, 1995)	*h* = −9→9
*T*_min_ = 0.395, *T*_max_ = 0.751	*k* = −9→9
6697 measured reflections	*l* = −17→17
3074 independent reflections	

### Refinement

**Table:**

Refinement on *F*^2^	Primary atom site location: structure-invariant direct methods
Least-squares matrix: full	Secondary atom site location: difference Fourier map
*R*[*F*^2^ > 2σ(*F*^2^)] = 0.028	Hydrogen site location: mixed
*wR*(*F*^2^) = 0.077	H atoms treated by a mixture of independent and constrained refinement
*S* = 1.20	*w* = 1/[σ^2^(*F*_o_^2^) + (0.0208*P*)^2^ + 0.8098*P*] where *P* = (*F*_o_^2^ + 2*F*_c_^2^)/3
3074 reflections	(Δ/σ)_max_ = 0.001
187 parameters	Δρ_max_ = 1.24 e Å^−^^3^
2 restraints	Δρ_min_ = −1.05 e Å^−^^3^

### Special details

**Table:**

Geometry. All e.s.d.'s (except the e.s.d. in the dihedral angle between two l.s. planes) are estimated using the full covariance matrix. The cell e.s.d.'s are taken into account individually in the estimation of e.s.d.'s in distances, angles and torsion angles; correlations between e.s.d.'s in cell parameters are only used when they are defined by crystal symmetry. An approximate (isotropic) treatment of cell e.s.d.'s is used for estimating e.s.d.'s involving l.s. planes.
Refinement. Refinement of *F*^2^ against ALL reflections. The weighted *R*-factor *wR* and goodness of fit *S* are based on *F*^2^, conventional *R*-factors *R* are based on *F*, with *F* set to zero for negative *F*^2^. The threshold expression of *F*^2^ > σ(*F*^2^) is used only for calculating *R*-factors(gt) *etc*. and is not relevant to the choice of reflections for refinement. *R*-factors based on *F*^2^ are statistically about twice as large as those based on *F*, and *R*- factors based on ALL data will be even larger.

### Fractional atomic coordinates and isotropic or equivalent isotropic displacement parameters (Å^2^)

**Table:**

	*x*	*y*	*z*	*U*_iso_*/*U*_eq_	
Cu1	0.54156 (8)	0.45571 (7)	0.28991 (4)	0.01103 (13)	
N1	0.6758 (5)	0.4606 (5)	0.4385 (3)	0.0113 (8)	
N2	0.3724 (5)	0.4527 (5)	0.1470 (3)	0.0108 (7)	
O1	0.6975 (4)	0.6960 (4)	0.3169 (2)	0.0158 (7)	
O2	0.4262 (4)	0.2003 (4)	0.2603 (2)	0.0133 (6)	
C1	0.7898 (6)	0.7701 (6)	0.4241 (3)	0.0113 (9)	
H1	0.7220	0.8632	0.4510	0.014*	
H2	0.9269	0.8337	0.4315	0.014*	
C2	0.7909 (6)	0.6272 (6)	0.4907 (3)	0.0101 (8)	
C3	0.8947 (7)	0.6571 (6)	0.5958 (4)	0.0149 (9)	
H3	0.9752	0.7751	0.6315	0.018*	
C4	0.8804 (7)	0.5124 (6)	0.6494 (3)	0.0146 (9)	
H4	0.9514	0.5299	0.7218	0.017*	
C5	0.7603 (6)	0.3425 (6)	0.5946 (3)	0.0128 (9)	
C6	0.6600 (6)	0.3186 (6)	0.4895 (3)	0.0115 (9)	
H5	0.5788	0.2016	0.4523	0.014*	
C7	0.2809 (6)	0.1401 (6)	0.1650 (3)	0.0133 (9)	
H6	0.1520	0.1080	0.1804	0.016*	
H7	0.3032	0.0268	0.1249	0.016*	
C8	0.2743 (6)	0.2825 (6)	0.0962 (3)	0.0117 (9)	
C9	0.1745 (6)	0.2415 (6)	−0.0109 (3)	0.0126 (9)	
H8	0.1095	0.1182	−0.0460	0.015*	
C10	0.1710 (6)	0.3826 (6)	−0.0657 (4)	0.0149 (9)	
H9	0.1049	0.3582	−0.1393	0.018*	
C11	0.2666 (6)	0.5611 (6)	−0.0107 (3)	0.0119 (9)	
C12	0.3689 (6)	0.5935 (6)	0.0947 (3)	0.0140 (10)	
H10	0.4377	0.7152	0.1312	0.017*	
Br1	0.72814 (7)	0.13936 (6)	0.66098 (3)	0.01408 (12)	
Br2	0.26359 (6)	0.75716 (6)	−0.08331 (3)	0.01288 (12)	
O3	0.7243 (5)	1.0111 (4)	0.2380 (3)	0.0190 (7)	
H11	0.709 (8)	0.913 (4)	0.254 (4)	0.029*	
H12	0.638 (6)	1.061 (7)	0.250 (4)	0.029*	

### Atomic displacement parameters (Å^2^)

**Table:**

	*U*^11^	*U*^22^	*U*^33^	*U*^12^	*U*^13^	*U*^23^
Cu1	0.0139 (3)	0.0090 (3)	0.0090 (3)	0.0016 (2)	0.0019 (2)	0.0016 (2)
N1	0.0058 (18)	0.0174 (19)	0.0100 (19)	0.0017 (15)	0.0021 (15)	0.0018 (17)
N2	0.0138 (19)	0.0119 (17)	0.0065 (18)	0.0035 (15)	0.0013 (15)	0.0032 (15)
O1	0.0173 (17)	0.0136 (16)	0.0147 (17)	−0.0008 (13)	0.0010 (13)	0.0075 (14)
O2	0.0165 (17)	0.0100 (14)	0.0112 (16)	−0.0008 (12)	0.0018 (13)	0.0036 (13)
C1	0.008 (2)	0.011 (2)	0.013 (2)	−0.0007 (17)	0.0015 (18)	0.0038 (19)
C2	0.009 (2)	0.010 (2)	0.010 (2)	0.0011 (16)	0.0032 (17)	−0.0027 (18)
C3	0.017 (2)	0.012 (2)	0.015 (2)	0.0028 (18)	0.0054 (19)	0.000 (2)
C4	0.020 (2)	0.015 (2)	0.010 (2)	0.0040 (19)	0.0053 (19)	0.003 (2)
C5	0.013 (2)	0.015 (2)	0.015 (2)	0.0045 (18)	0.0074 (19)	0.009 (2)
C6	0.014 (2)	0.009 (2)	0.013 (2)	0.0048 (17)	0.0062 (19)	0.0014 (19)
C7	0.013 (2)	0.012 (2)	0.011 (2)	0.0014 (18)	−0.0016 (18)	0.0031 (19)
C8	0.008 (2)	0.010 (2)	0.015 (2)	0.0002 (16)	0.0024 (18)	0.0007 (19)
C9	0.009 (2)	0.014 (2)	0.015 (2)	0.0014 (17)	0.0026 (18)	0.0044 (19)
C10	0.013 (2)	0.019 (2)	0.010 (2)	0.0011 (18)	0.0019 (18)	0.000 (2)
C11	0.015 (2)	0.015 (2)	0.010 (2)	0.0060 (18)	0.0066 (18)	0.0070 (19)
C12	0.016 (2)	0.013 (2)	0.016 (2)	0.0065 (18)	0.008 (2)	0.003 (2)
Br1	0.0183 (2)	0.0142 (2)	0.0112 (2)	0.00372 (17)	0.00498 (18)	0.00535 (19)
Br2	0.0155 (2)	0.0128 (2)	0.0122 (2)	0.00431 (17)	0.00462 (17)	0.00519 (18)
O3	0.023 (2)	0.0137 (16)	0.0228 (19)	0.0039 (14)	0.0071 (15)	0.0083 (16)

### Geometric parameters (Å, º)

**Table:**

Cu1—O1	1.882 (3)	C4—H4	0.9500
Cu1—O2	1.892 (3)	C5—C6	1.375 (6)
Cu1—N1	1.970 (3)	C5—Br1	1.891 (4)
Cu1—N2	1.991 (3)	C6—H5	0.9500
N1—C2	1.349 (5)	C7—C8	1.516 (5)
N1—C6	1.356 (5)	C7—H6	0.9900
N2—C8	1.331 (5)	C7—H7	0.9900
N2—C12	1.359 (5)	C8—C9	1.387 (6)
O1—C1	1.391 (5)	C9—C10	1.383 (6)
O2—C7	1.385 (5)	C9—H8	0.9500
C1—C2	1.499 (5)	C10—C11	1.390 (6)
C1—H1	0.9900	C10—H9	0.9500
C1—H2	0.9900	C11—C12	1.376 (6)
C2—C3	1.378 (6)	C11—Br2	1.890 (4)
C3—C4	1.396 (6)	C12—H10	0.9500
C3—H3	0.9500	O3—H11	0.797 (19)
C4—C5	1.387 (6)	O3—H12	0.817 (19)
			
O1—Cu1—O2	169.72 (13)	C6—C5—C4	120.2 (4)
O1—Cu1—N1	84.45 (13)	C6—C5—Br1	118.3 (3)
O2—Cu1—N1	93.40 (13)	C4—C5—Br1	121.6 (3)
O1—Cu1—N2	98.13 (13)	N1—C6—C5	120.6 (4)
O2—Cu1—N2	85.38 (13)	N1—C6—H5	119.7
N1—Cu1—N2	171.91 (14)	C5—C6—H5	119.7
C2—N1—C6	120.1 (4)	O2—C7—C8	113.1 (3)
C2—N1—Cu1	113.1 (3)	O2—C7—H6	109.0
C6—N1—Cu1	126.8 (3)	C8—C7—H6	109.0
C8—N2—C12	119.8 (4)	O2—C7—H7	109.0
C8—N2—Cu1	111.6 (3)	C8—C7—H7	109.0
C12—N2—Cu1	127.9 (3)	H6—C7—H7	107.8
C1—O1—Cu1	113.9 (2)	N2—C8—C9	122.0 (4)
C7—O2—Cu1	113.5 (2)	N2—C8—C7	114.5 (4)
O1—C1—C2	112.8 (3)	C9—C8—C7	123.5 (4)
O1—C1—H1	109.0	C10—C9—C8	119.1 (4)
C2—C1—H1	109.0	C10—C9—H8	120.5
O1—C1—H2	109.0	C8—C9—H8	120.5
C2—C1—H2	109.0	C9—C10—C11	118.4 (4)
H1—C1—H2	107.8	C9—C10—H9	120.8
N1—C2—C3	121.3 (4)	C11—C10—H9	120.8
N1—C2—C1	113.6 (4)	C12—C11—C10	120.2 (4)
C3—C2—C1	125.1 (4)	C12—C11—Br2	120.4 (3)
C2—C3—C4	119.4 (4)	C10—C11—Br2	119.4 (3)
C2—C3—H3	120.3	N2—C12—C11	120.5 (4)
C4—C3—H3	120.3	N2—C12—H10	119.7
C5—C4—C3	118.5 (4)	C11—C12—H10	119.7
C5—C4—H4	120.8	H11—O3—H12	109 (5)
C3—C4—H4	120.8		
			
O1—Cu1—N1—C2	6.8 (3)	C2—C3—C4—C5	−0.4 (6)
O2—Cu1—N1—C2	176.7 (3)	C3—C4—C5—C6	0.6 (6)
N2—Cu1—N1—C2	−102.2 (10)	C3—C4—C5—Br1	−178.9 (3)
O1—Cu1—N1—C6	−174.2 (3)	C2—N1—C6—C5	0.1 (6)
O2—Cu1—N1—C6	−4.3 (3)	Cu1—N1—C6—C5	−178.9 (3)
N2—Cu1—N1—C6	76.8 (11)	C4—C5—C6—N1	−0.5 (6)
O1—Cu1—N2—C8	165.7 (3)	Br1—C5—C6—N1	179.1 (3)
O2—Cu1—N2—C8	−4.6 (3)	Cu1—O2—C7—C8	11.3 (4)
N1—Cu1—N2—C8	−86.3 (10)	C12—N2—C8—C9	3.0 (6)
O1—Cu1—N2—C12	−5.0 (4)	Cu1—N2—C8—C9	−168.5 (3)
O2—Cu1—N2—C12	−175.2 (3)	C12—N2—C8—C7	−176.8 (3)
N1—Cu1—N2—C12	103.1 (10)	Cu1—N2—C8—C7	11.7 (4)
O2—Cu1—O1—C1	−91.0 (7)	O2—C7—C8—N2	−15.4 (5)
N1—Cu1—O1—C1	−12.7 (3)	O2—C7—C8—C9	164.8 (4)
N2—Cu1—O1—C1	159.6 (3)	N2—C8—C9—C10	−2.3 (6)
O1—Cu1—O2—C7	−114.6 (7)	C7—C8—C9—C10	177.5 (4)
N1—Cu1—O2—C7	167.8 (3)	C8—C9—C10—C11	−0.6 (6)
N2—Cu1—O2—C7	−4.2 (3)	C9—C10—C11—C12	2.8 (6)
Cu1—O1—C1—C2	15.9 (4)	C9—C10—C11—Br2	−179.8 (3)
C6—N1—C2—C3	0.2 (6)	C8—N2—C12—C11	−0.7 (6)
Cu1—N1—C2—C3	179.3 (3)	Cu1—N2—C12—C11	169.2 (3)
C6—N1—C2—C1	−178.9 (3)	C10—C11—C12—N2	−2.2 (6)
Cu1—N1—C2—C1	0.2 (4)	Br2—C11—C12—N2	−179.6 (3)
O1—C1—C2—N1	−10.3 (5)	N1—O1—N2—O2	−12.82 (13)
O1—C1—C2—C3	170.6 (4)	O1—N1—O2—N2	−13.32 (14)
N1—C2—C3—C4	0.0 (6)	N1—O2—N2—O1	11.80 (12)
C1—C2—C3—C4	179.0 (4)	O2—N1—O1—N2	11.96 (13)

### Hydrogen-bond geometry (Å, º)

**Table:**

*D*—H···*A*	*D*—H	H···*A*	*D*···*A*	*D*—H···*A*
O3—H11···O1	0.80 (2)	1.95 (2)	2.740 (4)	170 (6)
O3—H12···O2^i^	0.82 (2)	2.01 (2)	2.825 (4)	171 (5)

Symmetry code: (i) *x*, *y*+1, *z*.
